# The Relationship between Beliefs about Pain and Functioning with Rheumatologic Conditions

**DOI:** 10.1155/2012/206263

**Published:** 2012-06-26

**Authors:** Tracey Pons, Edward Shipton, Rodger Mulder

**Affiliations:** ^1^Department of Anaesthesia, University of Otago, P.O. Box 4345, Christchurch 8140, New Zealand; ^2^Department of Psychological Medicine, University of Otago, P.O. Box 4345, Christchurch 8140, New Zealand

## Abstract

Pain beliefs influence understanding of pain mechanisms and outcomes. This study in rheumatologic conditions sought to determine a relationship between beliefs about pain and functioning. Participants in Arthritis New Zealand's (ANZ) exercise and education programmes were used. Demographic data and validated instruments used included the Arthritis Impact Measurement Scale 2nd version-Short Form (AIMS2-SF) to measure functioning, and two scales of organic and psychological beliefs in Pain Beliefs Questionnaires (PBQ) to measure pain beliefs. 236 Members of ANZ were surveyed anonymously with AIMS2-SF and PBQ, with a 61% response rate; 144 responses were entered into the database. This study used *α* of 0.05 and a 1-*β* of 0.8 to detect for significant effect size estimated to be *r* = 0.25. Analysis revealed a significant relationship between organic beliefs scale of PBQ and functioning of AIMS2-SF, with an *r* value of 0.32 and *P* value of 0.00008. No relationship was found between psychological beliefs scale of PBQ and AIMS2-SF. Organic pain beliefs are associated with poorer functioning. Psychological pain beliefs are not. Beliefs might have been modified by ANZ programmes. Clinicians should address organic pain beliefs early in consultation. Causal links between organic pain beliefs and functioning should be clarified.

## 1. Introduction

Beliefs about pain are an emerging area of research in the biopsychosocial model of pain. Research shows that negative pain beliefs have a detrimental impact on patients' overall health, self-efficacy, and function [1]. With the intervention of a self-management programme of exercise and relaxation for arthritis sufferers, positive changes from negative pain beliefs correlate with improvement in self-efficacy, [2]. The experience of pain is a significant problem in sufferers with rheumatoid arthritis; it has been recently shown to be an important predictor for psychosocial health in general [3]. Furthermore, for rheumatoid arthritis, both the extent of the disease and the belief that pain could be capably managed have been found to impact on functioning [4, 5].

Beliefs have been defined as personally or culturally shared cognitive configurations [6]. These differ from attitudes that are defined as feelings about events. Beliefs are thoughts or mental appraisals and understanding of these events. These form the preexisting concepts about the nature of reality for the individual. These thoughts may be generalised or specific to certain contexts, mould the individual's perception of the environment, and shape the meaning of their experiences [7]. 

These thoughts can positively influence beliefs about the pain experience if, as perceived, there is control in managing the pain experience, confidence that the extent of harm and associated disability are not threatening, and expectations of recovery [8]. These thoughts can negatively influence beliefs about the pain experience if, as perceived, control is threatened and recovery is not possible [9]. The consequence can be emotional distress and catastrophising, as well as excessively negative and pessimistic beliefs and thoughts about the pain experience. Specific pain beliefs that contribute to poor compliance, motivation, and misunderstanding about pain have been identified [10]. These include catastrophising, limited perception of control over the pain experience, and emotional distress. Catastrophising has been shown to be associated with persistent pain; it is a predictor of poor outcomes in pain management interventions [5, 11]. Although catastrophising and emotional distress have common characteristics, it is difficult to separate them in the direction of effect. Thoughts about pain affect physical functioning and contribute to disability; physical functioning is predicted by the beliefs of physical capabilities and not by the experience of self-reported pain [12–14].

There is evidence that addressing negative pain beliefs in the management of persistent pain can affect treatment outcomes [4, 5, 15]. Negative pain beliefs can contribute to the transition from acute pain to persistent pain [6, 10].

It has been suggested that beliefs about persistent pain have two dimensions. These include organic pain beliefs (referring to the physiological pain experience indicating physical harm or threat to well-being) and psychological pain beliefs (referring to the internal influences and feelings affecting the experience of pain that can potentially threaten well-being); these dimensions are considered to accurately reflect the general population's perception of the pain experience [16]. Both of these can potentially influence the beliefs about pain control either positively (having personal control over the pain experience) or negatively (feeling helpless to manage the potential threat to their well being). 

The aim of this study was to determine the relationship between functioning and pain beliefs in patients with musculoskeletal pain or rheumatologic conditions. The primary hypothesis is that a relationship exists, whereby beliefs about persistent pain are associated with functioning. It is expected that a low score (less negative beliefs about pain) of the Pain Beliefs Questionnaire (PBQ) will be associated with better functioning in the Arthritis Impact Measurement Scale, second version, short form (AIMS2-SF). Likewise it is expected that a high score (greater negative beliefs about pain) of the PBQ will be associated with poorer functioning in the AIMS2-SF. The null hypothesis is that any observed relationship is simply due to chance.

## 2. Materials and Methods

**Figure 1:**
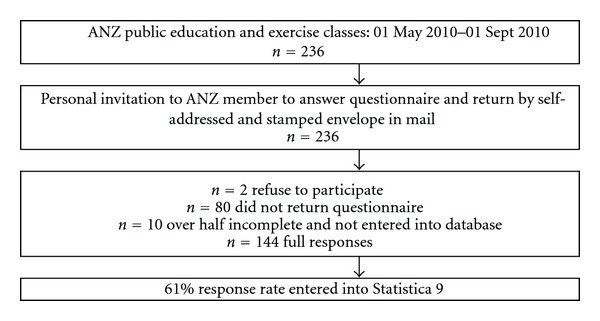
Flow chart: Method of data collection.

Members of Arthritis New Zealand (ANZ) who attended an exercise class, an individual education session with an ANZ Educator, or an education seminar were asked to voluntarily complete an anonymous questionnaire containing 72 questions. All 236 members taking part in the above activities were personally approached to participate in the study. Data was collected over six months, from 01 May 2010 to 31 October 2010. Care was taken to ensure that no participant answered a questionnaire twice. Most returned the answered questionnaire by mail in a preaddressed stamped envelope. Two participants declined to take part in the study and were not issued with a questionnaire. Eighty-two participants responded as willing to participate, were issued with questionnaires, but did not return them. Ten questionnaires were returned with less than half completed; these participants had missed out the middle pages as they filled them in. These were not entered into the database. Only if more than 50% of the questionnaire had been completed was it entered into the database. A total sample of 144 participants completed the questionnaires (a response rate of 61%). A flow chart (Figure 1) shows the details of the data collection. 

ANZ is a national voluntary organisation representing those with formal rheumatologic diagnoses. It functions as a charitable trust with modest government funding. Activities include member education on arthritis and pain management, exercise classes, and seminars [17]. Ethical approval was obtained from the Upper South Island A Regional Ethics committee (reference number URA/10/04/026). The process followed in this study was in accordance with the Helsinki Declaration of 1975, as revised in 1983.

The questionnaires consisted of the PBQ and the AIMS2-SF. In addition further questions were asked about the following: gender; duration of pain experience; age; number of months of physiotherapy intervention; type and frequency of regular exercise; enjoyment and confidence with exercise; ability to stop exercise; previous physical and athletic ability; anger about pain; duration of membership with ANZ; ethnicity (as defined by Statistics New Zealand) [18], education level; rheumatologic diagnosis; number of months off work; smoking habit; medication use. 

### 2.1. Instruments to Measure Beliefs about Pain

Several questionnaires are used to measure the beliefs about pain. The Pain Beliefs Questionnaire (PBQ) is a validated and reliable questionnaire that taps into the two dimensions of pain beliefs (organic and the psychological beliefs) and was developed to describe these beliefs about pain [16, 19, 20]. It was chosen for this study as it allowed for differentiation between these two separate dimensions of pain beliefs, was easy to administer, and was not time consuming or lengthy for patients. The questions of the PBQ are listed in Table 1.

The internal consistency of each scale of the PBQ has been shown to be 0.73 for the organic scale and 0.70 for the psychological scale [16]. A Likert scale of 1–5 was used to measure each question. The organic scale consists of eight questions; these measure the extent of the belief that that personal control of the pain is impossible (due to physical harm or injury believed to be associated with the pain experience). The psychological scale consists of four questions; these measure the extent of the belief that personal control of the pain is linked to the emotional feelings about the pain experience. The higher the summed score on the PBQ, the greater is the belief that harm and emotional feelings negate personal control of the pain experience. 

There are other seven questionnaires that measure beliefs about pain; these were excluded, as they were either too lengthy, not specific to rheumatologic conditions, or outside the domain of this study. They were as follows: the Illness Perceptions Questionnaire (revised) [21]; the Back Beliefs Questionnaire [22]; the Fear Avoidance Beliefs Questionnaire (FABQ) [23]; the Survey of Pain Attitudes (SOPA) [24]; the Pain Beliefs and Perceptions Inventory (PBAPI) [25]; the Pain Cognitions Questionnaire [26]; the Cognitive Risk Profile [27].

### 2.2. Instruments to Measure Functioning

Research has shown that self-report questionnaires are valid measures for the assessment of functioning [28]. There are a wide variety of functioning measures that can be determined using questionnaires. The AIMS2-SF questionnaire was chosen for this project as (i) it is appropriate for the population sample of rheumatologic conditions; (ii) it is an instrument that measures upper limb, lower limb, and whole body functional movements (unlike most other instruments that do not); (iii) it is a suitable and reliable instrument for measuring disability in personal care for those suffering rheumatologic conditions [29]; (iv) the shortened version of the revised AIMS2 has been validated and is reliable with similar psychometric properties; it is easy to administer and is useful for assessing functioning status with other persistent conditions besides arthritis [30]. The AIMS2-SF is appropriate to use in this study, as it is symptom specific as well as able to reliably enquire about the broader areas of functioning. 

The AIMS2-SF was developed in concordance with the World Health Organisation's (WHO) International Classification of Functioning, Disability, and Health (ICF) [31] and has been widely used for measuring functioning in rheumatologic diseases [29, 32–34]. The scale includes a broad domain of functional ability with the combination of the five second-order scores. The second-order scores consist of (i) physical aspects (mobility level, walking, bending, arm/hand/finger function, self-care, household tasks); (ii) affect (level of tension and mood); (iii) self-reported pain; (iv) social interaction (social activity, support from family); (v) work. 

The Health Assessment Questionnaire is another instrument applied to measuring functioning in rheumatologic conditions [35–37]. It was not chosen for this study as the AIMS2-SF covers broader areas of functioning, including work and affect. Fourteen other possible questionnaires considered for this study were excluded; the reasons for this varied. For example, they were not inclusive of both upper and lower limb functioning, addressed back pain only, addressed pain experiences as “sickness,” or restricted the age group involved. These other questionnaires were as follows: the Oswestry Disability Index [38, 39]; the Roland Morris Disability Scale [40]; the Acute Low Back Pain Screening Questionnaire [41, 42]; the Vermont Disability Prediction Questionnaire [42]; the Screening Questionnaire for predicting outcome in acute and subacute back pain [42]; the Orebro Musculoskeletal Pain Questionnaire [43, 44]; the Chronic Pain Coping Inventory [45]; the International Physical Activity Questionnaire [46]; the Pain Disability Index [47]; the West Haven-Yale Multidimensional Pain Inventory (WHYMPI) [48]; the Sickness Impact Profile [49–51]; the Physical Activity Scale for the elderly (PASE) [52]; the Quebec Back Pain Disability Scale [53]; the Functional Disability Inventory [54].

The factorial validity of the AIMS2-SF has been verified [29]. The higher the total summed AIMS2-SF score, the poorer the functioning. The highest possible score is 120; this indicates exceptionally poor functioning. Such a person is highly dependent on assistance for all daily activities, has a high pain experience, and has low mood and poor social support. On the other hand, a low score indicates greater functioning with both upper and lower limbs, with whole body tasks, and with independence. 

### 2.3. Analysis

The analysis for this study was to use simple linear regression between the sum of the AIMS2-SF and the sum of each of the two scales of the PBQ. This study used an *α* of 0.05, and a 1-*β* of 0.8 to detect for significant effect size, estimated to be *r* = 0.25.

## 3. Results

### 3.1. Demographic Data

A sample of 122 participants had been calculated for the effect size “*r*” of 0.25 [55]; 144 responses (61% response rate) were entered into Statistica 9 for statistical analysis. The demographic data for all participants is summarised in Table 2 as predominant percentages of the full data set. 

The mean age of the participants was 65 years (SD 11 years, 8 months). Eighty-five per cent of the participants were females. The mean for participants' duration of pain experience was 130 months (almost 11 years of pain). The standard deviation remained large. Most participants in this study were new members of Arthritis New Zealand (ANZ) where 61% of participants had joined the organisation in the past three years. 

Eighty-eight per cent of all participants were classified as New Zealand European/Pakeha. The predominant group (36%) suffered from osteoarthritis. Other diagnoses included rheumatoid arthritis (18%), fibromyalgia (15%), no diagnosis given (12%), “undefined” arthritis (7%), inflammatory arthritis (2%), and temporal arteritis, systemic lupus, and other diagnoses (1%). Fifty-five per cent of participants received no formal qualification other than schooling. 

### 3.2. Questionnaire Data for Primary Hypothesis Testing

#### 3.2.1. AIM2-SF Scores

The AIMS2-SF has second-order scores consisting of (i) physical aspects (mobility level, walking, bending, arm/hand/finger function, self-care, household tasks); (ii) affect (level of tension and mood); (iii) self-reported pain; (iv) social interaction (social activity, support from family); (v) work. Each of these second-order scores was summed and correlated with the organic scale of the PBQ. The highest correlation was found with the work subscale, *r* = 0.36 and *P* = 0.03. The physical activity, affect and pain experience correlated similarly with *r* = 0.34 and *P* = 0.00003. However social activity had a high correlation, *r* = 0.69 but with no significance *P* = 0.4. This data is outlined in Table 3.

The participants showed a reasonable distribution of summed AIM2-SF scores. A higher score is associated with a poorer functioning; a lower score is associated with greater functioning. Fourteen per cent of participants scored between 70 and 100, indicating greater disability; 9% of participants scored below 40, indicating excellent functional health. The mean score was 57 with a standard deviation of 12 points.

#### 3.2.2. The Duration of Pain Experience

The mean for the participants' duration of pain experience was 130 months (almost 11 years) of pain. The standard deviation is large with 125 months (10.4 years). The longest duration of pain recorded by a participant was 55 years and the lowest recorded was 2 months; 51% of participants had experienced 8 years or more of pain. 

#### 3.2.3. Beliefs Measured on the Organic Scale

The mean score in this project was 24 with a standard deviation of 6.

#### 3.2.4. Beliefs Measured on Psychological Scale

The psychological scale of the PBQ mean score was 13 with a standard deviation of 4. 

The data for the primary hypothesis testing is outlined in Table 4.

### 3.3. Hypothesis Testing for the Relationship of the PBQ and the AIMS2-SF

Simple linear regression analysis was applied with each of the subscales sum of the PBQ and the AIMS2-SF. The data showed two different relationships of scatter plot with simple linear regression for each subscale of the PBQ with the AIMS2-SF. The relationship between the organic beliefs subscale reached significance (*P* = 0.0002) with a modest correlation coefficient (*r* = 0.32). The Spearman Rank Order Correlation is 0.3297 for the relationship between the two variables “sum of AIMS” versus “sum of BELIEFS” and is significant at *P* < 0.05. 

**Figure 2:**
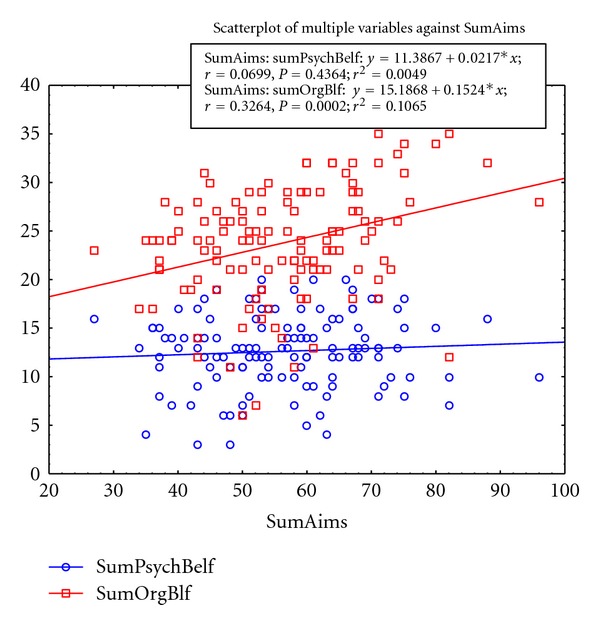
Scatterplot with linear regressions of the sum of AIMS2-SF disability with both the psychological subscale of the PBQ and the organic subscale of the PBQ.

The relationship for the psychological scale showed no significance (*P* = 0.4) and no correlation (*r* = 0.06). The scatter plot with the simple linear regression is shown in Figure 2.

The primary hypothesis tests confirmed that organic beliefs about persistent pain are associated with functioning. A low score (less negative beliefs about pain) of the organic scale of the PBQ was associated with better functioning in the AIMS2-SF. A high score (greater negative beliefs about pain) of the organic scale of the PBQ was associated with poorer functioning in the AIMS2-SF. The psychological scale of the PBQ showed no relationship with disabled functioning. 

## 4. Discussion

### 4.1. Outcomes Achieved

The primary hypothesis in this study is confirmed. There is a significant relationship between organic beliefs about pain and functioning. This was shown for participants in the Arthritis New Zealand exercise classes and education programmes. The sample consisted of patients with rheumatologic conditions with pain for 8 years and more (51%) and ranged from 22 to 91 years in age (mean age of 65 years; SD 11.7 years). 

The participants in this study would have benefited from the education and support provided by ANZ for managing their pain and improving their exercise and functioning. It is not possible in this study to measure the modifying extent due to the participants' involvement with the ANZ programmes. This correlation is nevertheless present despite the membership and participation with ANZ. 

Data from this study show that patients with rheumatologic conditions who have had pain for more than 8 years and have a high score of organic beliefs are likely to exhibit poor functioning. High organic beliefs are the beliefs that the pain experience indicates harm or a threat to well being. The converse is also true. This strong evidence dissects organic beliefs from psychological beliefs for their respective influence on functioning. Furthermore, this study's contribution is consistent with the current literature that the transition from acute to persistent pain is associated with cognitive-affective factors (such as negative beliefs and low self-efficacy) [56–59]. 

Beliefs contribute to the formation of an individual's perception of reality. Pain beliefs are thoughts about the perceived control of the pain experience. These include the extent to which the pain experience is perceived to be harmful, the perceived disability associated with the pain experience, and the expectations of recovery [60]. Those who have a low score of organic pain beliefs are likely to have better functioning. This confirms the literature that organic beliefs about pain influence catastrophising and functional disability [60]. 

Organic pain beliefs contribute to the perception that pain is harmful and that control of pain is not possible; this weaves a common thread with catastrophising. Catastrophising is widely known to influence functional disability; it influences the perception of harm and disability associated with the pain experience and influences expectations of recovery [61]. It would make sense that high organic pain beliefs should have a relationship with poor functioning. Pain catastrophising plays a significant role in the experience of pain and predicts the persistence of pain [62–65]. This raises the possibility that organic pain beliefs have similar effects to catastrophising in influencing outcomes in those with persistent pain. The most recent literature about catastrophising shows that the relationship between coping, pain adjustment, and catastrophising is still not fully understood [66]. 

This study raises the differences between the two dimensions of pain beliefs (organic and psychological) and their relationship with functioning. Psychological and occupational factors can also contribute to persistence of the pain experience [10, 67–71]. In this sample, it is shown that psychological beliefs, the beliefs about the internal influences and feelings affecting the experience of pain, do not have any relationship with functioning. This is in contrast to systematic reviews that have shown psychological and occupational factors to have the highest reliability for prognostic factors contributing to persistence of the pain experience [10, 67–70]. There is a growing body of research supporting the model that the transition from acute to persistent pain is associated with serious life stressors including cognitive-affective factors [56]. When the PBQ was first developed it was noted that patients with persistent pain were more likely to support the organic beliefs items, while nonpatients were more likely to support the psychological beliefs items [16].

### 4.2. Strengths and Weaknesses

The strength of this study is the novel association of the two instruments, the AIMS2-SF and the PBQ for rheumatologic conditions, and the evidence that this relationship is consistent with international research. A correlation of these two instruments has not yet been applied. A further strength is the specific focus on ANZ with a moderate sample size within the larger population of rheumatologic conditions. 

Several factors might account for the fact that the psychological beliefs do not show a relationship with functioning. The psychological beliefs scale has only 4 questions, in contrast to the 8 in the organic scale. It is perhaps a limitation of this questionnaire that the psychological beliefs are less featured or that specifically it is the organic beliefs rather than psychological beliefs that contribute to the persistent pain affecting functioning. The ANZ programmes and input could have influenced the data acquired. Sixty-one per cent of participants had joined the ANZ organisation over the previous three years. The functioning and psychological beliefs of responders may have been modified through their previous participation in the seminars and exercise groups, whereas the organic beliefs may not have been modified. 

Since this study was not longitudinal in design, the direction of effects between the organic pain beliefs and functioning could not be determined. Furthermore, the sample group is limited to the members of ANZ in a small geographical area; these findings would need to be confirmed in wider areas. Nonresponders were 35% of those approached; there remains the possibility that their beliefs about pain and functioning may have differed from those of the responders. 

There was a high percentage (85%) of female participants in the sample. Men might have different beliefs about pain and express them differently; their levels of functional ability associated with persistent pain might differ from those of women [72, 73]. The study only captured 4% of Maori participants, due to Christchurch being on the South Island with fewer Maori and Pacific Island populations. New Zealand health statistics generally show poorer health and socioeconomic status for the Maori and Pacific Island populations. 

### 4.3. Implications for Clinical Practitioners

Research outcomes for evidence-based medicine are becoming more important for everyday clinical decisions and health funding. This study provides evidence that organic beliefs and functioning are indeed related. It is recommended that clinicians ask their patients about pain beliefs. It would be sensible to address organic pain beliefs early in their consultations, as patient beliefs about pain are influenced by interactions with health professionals [74–76]. The way to do this may be to make use of target questions to reveal underlying organic beliefs possibly contributing to poor functioning. For example, “*Do you believe that it is impossible for you to control your pain*,” or “*do you believe that your pain means that there is something permanently wrong with your body?*” Patients with rheumatologic conditions who respond in the affirmative should be encouraged to modify their organic beliefs. Information and education based on a biopsychosocial model have been shown to be effective in modifying beliefs, improving outcomes and in treatment compliance [77].

### 4.4. Implications for Future Research

These data show that organic beliefs and functioning are significantly related. However, psychological beliefs and functioning are not. Future research should determine the direction of this effect. The causal links between organic pain beliefs and functional ability for rheumatologic conditions could be further explored and relationships between organic beliefs and catastrophising investigated. The influences of organic and psychological pain beliefs with functioning in other medical conditions can be probed. Possible cultural and gender factors influencing pain beliefs in different populations (e.g., Maori and Pacific Islanders, refugees, other minority groups in New Zealand) could be considered as well. 

This study confirmed a relationship between organic beliefs about pain and functioning (as shown by participants in the Arthritis New Zealand exercise classes and education programmes). As previously stated, the functioning and beliefs of responders may have been modified through their previous participation in the seminars and exercise groups. Particulary, how the programmes of ANZ influence and modify beliefs about pain or improve functioning as well as their variability in efficacy across the different geographic locations requires more investigation. 

The instruments used in this study might not have been able to detect a relationship between the psychological beliefs about pain and functioning. On the other hand, rheumatologic conditions might have psychological features about their belief systems that differ from other population groups with pain. The influence of psychological feelings on the experience of pain in rheumatologic conditions should be explored as well. 

## 5. Conclusion

This study confirmed a relationship between organic beliefs about pain and functioning for participants in the ANZ exercise classes and education programmes. The sample had a mean age of 65 years (SD 11.7 years); fifty-one per cent of participants had experienced more than 8 years of pain, and 61% had joined ANZ in the last three years. Organic pain beliefs (beliefs that the pain experience indicates harm or threat to well being) are significantly associated with poorer functioning. This was confirmed by linear regression with an *r*-value of 0.32 (where *α* was 0.05 and 1-*β* was 0.8). Psychological pain beliefs (beliefs about the internal influences and feelings affecting the experience of pain) are not associated with functioning. In this study that beliefs could have been modified by the ANZ programmes. 

When functioning is impaired organic beliefs about pain need to be addressed in the management of rheumatologic conditions. It is recommended that clinicians ask their patients about pain beliefs and address organic pain beliefs early in their consultations. Patients with rheumatologic conditions who respond positively to questioning should be encouraged to modify their organic beliefs. 

Future research in rheumatologic conditions will determine the possible relationship between organic beliefs and catastrophising, as well as establish the causality between pain beliefs and functioning. 

## Figures and Tables

**Table 1:** PBQ organic and psychological pain beliefs of the PBQ.

Organic pain beliefs
Persistent pain is the result of damage to tissues of the body
Physical exercise makes the persistent pain worse
It is impossible to do much for oneself to relieve persistent pain
Persistent pain is a sign of illness
Experiencing persistent pain is a sign that something is wrong with the body
It is impossible to control your own persistent pain
Being in persistent pain prevents you from enjoying hobbies and social activities
The amount of persistent pain is related to the amount of damage

Psychological pain beliefs
Being anxious makes persistent pain worse
Thinking about persistent pain makes it worse
When relaxed persistent pain is easier to cope with
Feeling depressed makes persistent pain seem worse

**Table 2:** Demographic predominant percentage data.

Ethnicity	NZ European 88%
Diagnosis	Osteoarthritis 36%
	Rheumatoid arthritis 17%
	Fibromyalgia 15%
	Unknown 13%
	Polymyalgia rheumatica 8%

Gender	Female 85%

Age	Mean: 65 years
	Std Dev 11 years, 8 months
	Highest recorded data: 91 years
	Lowest recorded data: 22 years

Educational level	School only, 55%

Physiotherapy intervention for pain	54%

Membership with ANZ within last 3 years	61%

Smoking	93% non smokers

Time off work because of pain experience	32%

Number of years with pain	51% greater than 8 years
	Mean: 130 months (almost 11 years)
	Std dev: 125 months (10.4 years)
	Highest recorded data: 55 years
	Lowest recorded data: 2 months

**Table 3:** Second-order AIMS2-SF scales and correlation with PBQ organic scale.

AIMS2-SF subscale	Mean	Std dev	Median	Correlation *r* with PBQ organic scale	*P* (linear fit)
Physical activity	295.2	42.3	297	*r* = 0.34	*P* = 0.00003
Affect	10.8	4.2	10	*r* = 0.33	*P* = 0.00004
Pain	9.8	2.8	10	*r* = 0.34	*P* = 0.00003
Social	11.5	2.9	12	*r* = 0.69	*P* = 0.4
Work	3.1	1.7	2	*r* = 0.36	*P* = 0.03

**Table 4:** Data for primary hypothesis testing.

	Mean	Std dev	Max score	Min Score	Highest possible score
AIMS2-SF	57.3	12.7	101	27	130
PBQ organic belief scale	23.8	5.8	37	6	40
PBQ psychological belief scale	12.6	3.9	20	2	20
Duration of pain (years)	11.1	10.9	—	—	—
